# A Saliency Guided Semi-Supervised Building Change Detection Method for High Resolution Remote Sensing Images

**DOI:** 10.3390/s16091377

**Published:** 2016-08-27

**Authors:** Bin Hou, Yunhong Wang, Qingjie Liu

**Affiliations:** State Key Laboratory of Virtual Reality Technology and Systems, Beihang University, Beijing 100191, China; houbin@buaa.edu.cn (B.H.); yhwang@buaa.edu.cn (Y.W.)

**Keywords:** change detection, remote sensing, extended morphological attribute profiles, saliency, morphological building index

## Abstract

Characterizations of up to date information of the Earth’s surface are an important application providing insights to urban planning, resources monitoring and environmental studies. A large number of change detection (CD) methods have been developed to solve them by utilizing remote sensing (RS) images. The advent of high resolution (HR) remote sensing images further provides challenges to traditional CD methods and opportunities to object-based CD methods. While several kinds of geospatial objects are recognized, this manuscript mainly focuses on buildings. Specifically, we propose a novel automatic approach combining pixel-based strategies with object-based ones for detecting building changes with HR remote sensing images. A multiresolution contextual morphological transformation called extended morphological attribute profiles (EMAPs) allows the extraction of geometrical features related to the structures within the scene at different scales. Pixel-based post-classification is executed on EMAPs using hierarchical fuzzy clustering. Subsequently, the hierarchical fuzzy frequency vector histograms are formed based on the image-objects acquired by simple linear iterative clustering (SLIC) segmentation. Then, saliency and morphological building index (MBI) extracted on difference images are used to generate a pseudo training set. Ultimately, object-based semi-supervised classification is implemented on this training set by applying random forest (RF). Most of the important changes are detected by the proposed method in our experiments. This study was checked for effectiveness using visual evaluation and numerical evaluation.

## 1. Introduction

Timely and accurate change detection of the land cover (LC) and land use (LU) information is extremely important for applications, such as monitoring environmental changes and resource management. Image change detection (CD) involves the analysis of two registered images acquired over the same geographical area at different times in order to identify differences in the state or type of physical materials on the Earth’s surface [1]. Remote sensing (RS) data have become a major source for CD studies due to their high temporal frequency and wide selection of spectral and spatial resolutions. CD methods could be categorized as either supervised or unsupervised according to the nature of data processing. Supervised methods require an appropriate training set, which makes them a difficult and expensive task. Unsupervised methods without any prior information are more widely used and studied.

Over the past few years, a variety of different unsupervised CD algorithms have been proposed [2,3,4], in which pixel-based pre-classification CD techniques have been developed mainly including: (a) image differencing [5]; (b) image ratioing; (c) vegetation index differencing; (d) change vector analysis (CVA) [6]; (e) principal component analysis (PCA) [7]; and (f) expectation-maximization (EM) algorithm [5]. Differing from detecting simple binary changes, pixel-based post-classification comparison can get detailed “from-to” change information [8,9,10,11]. Nevertheless, most of the above CD techniques mainly focus on low and medium spatial resolution images.

In recent years, with the increasing availability of high resolution (HR) remote sensing images, it is possible to identify detailed changes occurring at the level of ground structures, of which buildings are paid the most attention. The emergence of high-resolution Earth observation data brings a huge challenge to traditional information extraction techniques. Conventional pixel-based CD techniques are considered ineffective for HR remote sensing data because of high intraclass variability and low inter-class variability on these data. Another important limitation is the difficulty of modeling the contextual information. To solve these problems, spatial dependence among neighboring pixels, e.g., object, textural or structural-based image description, have been utilized in CD [2].

The object-based change detection (OBCD) techniques [12,13,14] have been shown to reduce the effects of geo-referencing, higher spectral variability and acquisition characteristics. Im et al. [12] proposed an object-based change detection method based on image segmentation and object/neighborhood correlation image analysis. The method was based on the fact that the pairs of brightness values from the same geographic area (e.g., an object) between bi-temporal image datasets tend to be highly correlated for unchanged and uncorrelated for changes. Bovolo [15] proposed a novel parcel-based context-sensitive CD technique for very high resolution (VHR) remote sensing images. CD was achieved by applying a multilevel CVA to each pixel. Huo et al. [16] proposed a fast object-level change feature extraction and classification. They improved the accuracy and the degree of automation by dynamically adjusting the training samples and gradually tuning the separating hyperplane in the support vector machine (SVM). Dalla Mura et al. [17] integrated morphological filters and the CVA techniques for high resolution image change detection, demonstrating greater accuracy than traditional pixel-based CVA. Falco et al. [18] extracted the geometrical features related to the structures within the scene at different scales for CD using a multiresolution contextual transformation performed by attribute profiles (APs). Huang et al. [19,20] investigated urban building change detection. They combined several pieces of building information, including morphological building index (MBI), spectral and shape conditions for multitemporal high-resolution images. Ding et al. [21] proposed a sparse hierarchical clustering approach for VHR image CD. They stacked bi-temporal multiscale center-symmetric local binary pattern features and learned a tree-structured dictionary. Zhong et al. [22] improved the traditional automatic change detection method with pulse-coupled neural networks (PCNN). They combined PCNN with the normalized moment of inertia (NMI) feature for high spatial resolution imagery. Robertson and King [23] compared pixel- and object-based classification in land-cover change mapping. They revealed that the object-based approach depicted change information more accurately.

In spite of some efforts having been made to develop high-resolution CD techniques, how to characterize discriminative object-based features for the extraction of sophisticated geospatial information is difficult. A widely-used feature is the mean value of the pixels inside the object, the main limitation of which is obvious, for it only considers the spectral values of pixels to construct the object feature while ignoring the texture information and losing much of the spectral information. Modeling contextual information using the local adaptive neighborhood of pixels not only exploits the spectral characteristics, but also considers the spatial context. However, the different scales of the observation window could not conform to the various sizes of the true geographical objects.

In this paper, an object-based building CD approach combining pixel-based post-classification is proposed to address the aforementioned problems. We propose a novel framework for urban building CD of HR remote sensing images. Pixel-based post-classification of this method is based on recently-developed extended morphological attribute profiles (EMAPs) [24], which is able to characterize spatial features by performing a multiresolution filtering of the multitemporal images. After that, the hierarchical fuzzy histogram is constructed for each region segmented using simple linear iterative clustering (SLIC) [25]. Furthermore, saliency [26] and the MBI [20] map generated from the difference image by object-based CVA are utilized to get a pseudo training set as the input of the random forest (RF) classifier. The experimental results indicate that the proposed approach is effective and feasible.

This paper consists of four sections. The next section describes our approach, including: (1) feature extraction and representation; (2) super-pixel segmentation and hierarchical fuzzy histogram construction; (3) saliency and MBI for final change detection. In Section 3, we present the used datasets, the experimental results and the discussion. The conclusions are drawn in Section 4.

## 2. Methodology

**Figure 1:**
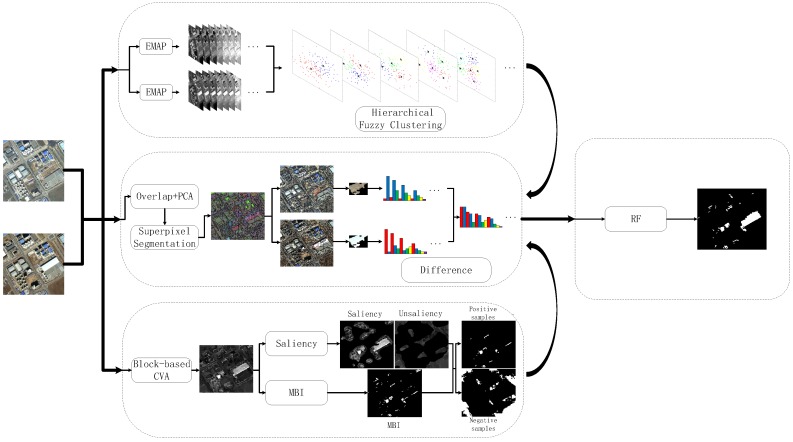
Flowchart of the proposed method. EMAP, extended morphological attribute profile; CVA, change vector analysis; MBI, morphological building index.

In this section, we introduce the proposed method, which is composed of the following three steps. First, we present how to characterize the spectral-spatial information of HR images by using the multi-level, multi-attribute approach-based EMAPs. Following that, fuzzy clustering is applied to EMAP feature vectors for each pixel. Then, we extract image-objects using SLIC segmentation, and a hierarchical fuzzy histogram is generated for each object. Finally, saliency detection is applied, and the MBI feature is employed to obtain the final object level CD. The most salient building regions serve as the training sets, called pseudo training sets, for the RF classifier. The general scheme is shown in Figure 1.

### 2.1. Feature Extraction and Representation

The change feature should be discriminative to distinguish the different distributions of HR images. The simple spectral feature has difficulty in satisfying the aforementioned requirements even if the spectral mean of pixels within a certain neighborhood is used. Although object extraction by segmentation has the advantage of being able to make defining window size and shape more flexible, an important challenge with image-object CD is how to extract the feature, which is not only robust to lighting condition variations, seasons changes and sensor noise, but also ideal to represent the corresponding object. The spatial organization between pixels is considered to be crucial. As a consequence, a feature extraction method that combines spectral with spatial information is required to put forward for reducing labeling uncertainty. Moreover, spatial information provides additional discriminant information related to the shape and size of different structures. One way to extract spatial information by using a crisp neighborhood system is considered as the Markov random field (MRF) modeling [5], which is a powerful tool for incorporating spatial and contextual information into each pixel. Its limitations are that the standard neighborhood system may not contain enough samples to characterize the object of interest, and a larger neighborhood system leads to intractable computational problems. One possible way to solve these problems is to utilize different types of segmentation methods, whereas they encounter the uncertainty of the object boundary.

Another set of methods that can extract spatial information by using an adaptive neighbor system is based on morphological filters, which can avoid the above problems well. Pesaresi and Benediktsson [27] used morphological transformations to build a morphological profile (MP) and introduce them to HR images. They performed a multiscale analysis by computing an anti-granulometry and a granulometry, i.e., a sequence of openings and closings by reconstruction with an structuring element (SE) of increasing size, applied to a scalar image. MPs computed with a compact SE (e.g., square, disk, etc.) can be used for modeling the size of the image-objects. Furthermore, the concept of MPs was successfully extended to handle hyperspectral images, resulting in the extended morphological profiles (EMPs), which are obtained by computing an MP on each of their first few components [28,29]. Multiscale processing based on morphological filters (e.g., by MPs and EMPs) has been proven to be an effective strategy for extracting informative spatial features from the analyzed images. However, primary limitations lie in the following points: (1) the shape of SEs is fixed; and (2) SEs cannot represent the information of the gray-level characteristics of the regions. To overcome this, the morphological attribute profiles (APs) have been proposed as the extension of the MPs and provide a multilevel characterization of an image by using the sequential application of morphological attribute filters (AFs), which can be considered for modeling different specifications of the structural information [24]. AFs process an image only considering its connected components, which are proven to be efficient for modeling structural information in VHR images. The use of different attributes leads to the generation of extended multi-attribute profiles (EMAPs) [24]. Next, we will give the detailed description.

Morphological attribute opening and closing are morphological AFs [30], which are connected operators processing an image by considering only its connected components. The common connected components are 4- and 8-connected, where a pixel is considered adjacent to four or eight of its neighboring pixels, respectively. For a grayscale image *f*, the set of connected components can be obtained by representing the image as a stack of binary images generated by thresholding it at each gray-level value. AFs preserve or merge the connected components *C* based on a predicate *P* if a given attribute A is greater/lower than a predefined threshold value *λ*, i.e., P(C)=A(C)≥λ(A(C)≤λ). If *P* is met, the region is preserved; otherwise, it is merged to the adjacent region with a closer gray-level value. An AP is obtained by the sequence of attribute thinning and thickening transformations with a series of progressively stricter criteria. More formally, an AP is defined as follows:

(1) $$AP\left(f\right)=\left\{\phi^{P_{\lambda_{L}}}\left(f\right),\phi^{P_{\lambda_{L-1}}}\left(f\right),...,\phi^{P_{\lambda_{1}}}\left(f\right),f,\gamma^{P_{\lambda_{1}}}\left(f\right),...,\gamma^{P_{\lambda_{L-1}}}\left(f\right),\gamma^{P_{\lambda_{L}}}\left(f\right)\right\}$$

where $\gamma^{P}\left(f\right)$ and $\phi^{P}\left(f\right)$ denote an attribute thickening and thinning, respectively. The considered criteria are increasing, i.e., $P(C_{j})=true$ when also $P(C_{i})=true$ for any $C_{j}\subseteq C_{i}$. The original image *f* is also contained in the profile since it can be considered as the level zero (i.e., $\phi^{P_{\lambda_{0}}}\left(f\right)=\gamma^{P_{\lambda_{0}}}\left(f\right)=f$). Different information can be extracted from the multi-level characterizations of the image by AP. AP can be efficiently computed by representing the input image as a rooted hierarchical tree of the connected components of the image, i.e., the max-tree algorithm [31]. The EAP is obtained by stacking the AP on each of the first *k* principal components (PCs), which are obtained by applying feature extraction on the multi/hyperspectral image as the following equation:

(2) $$EAP\left(f\right)=\{AP\left(f_{1}\right),AP\left(f_{2}\right),...,AP\left(f_{k}\right)\}$$

During the concatenation of different attributes, the EMAP is obtained and given mathematically by:

(3) $$EMAP\left(f\right)=\{EAP_{A_{1}}\left(f\right),EAP_{A_{2}}^{\prime }\left(f\right),...,EAP_{A_{m}}^{\prime }\left(f\right)\}$$

where $EAP_{A_{i}}$ is an EAP built with a set of predicates *P* evaluating *m* different kinds of attributes $A_{i}\left(i\leq m\right)$ and $EAP^{\prime }=EAP\backslash {\left\{f_{i}\right\}}_{i=1,...k}$ in order to avoid redundancy since the original image *f* is presented in all of the EAP. The following attributes have been widely used in the literature for EMAP:

- area of the region (a measure of the size of the regions, denoted as ‘*a*’);
- standard deviation (a measure of the homogeneity of the regions, denoted as ‘*s*’);
- diagonal of the box bounding the regions (another measure of the size of the regions, denoted as ‘*d*’);
- moment of inertia (a measure of the elongation of the regions, denoted as ‘*i*’).

**Figure 2:**
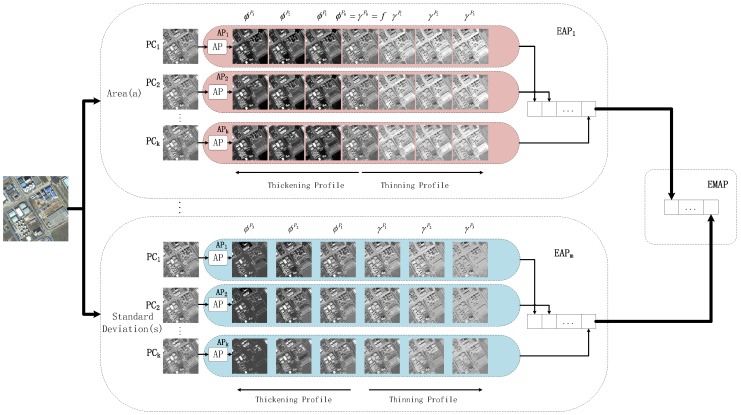
General architecture of EMAP.

APs, while considering the above attribute measures, perform a contextual analysis of the image, which permits a richer description of the regions since the filtering is performed according to measures of their spectral, spatial, textual and other characteristics. While the APs can be constructed on the basis of different attributes, generally only the two attributes of area and standard deviation are used, since they not only can be adjusted in an automatic way, but also are well related to the object hierarchy of the images. In addition, they can model the spatial information considerably, while other attributes (i.e., diagonal of the box bounding the region and the moment of inertia) cannot add significant improvement to classification accuracy. With regard to $\lambda_{a}$ for the area attribute, the resolution of the image should be taken into account [32]. The automatic scheme of the attribute area is given as follows:

(4) $$\lambda_{a}\left(PC_{i}\right)=\frac{1000}{v}\left\{a_{min},a_{min}+\delta_{a},a_{min}+2\delta_{a},...,a_{max}\right\}$$

where $a_{min}$ and $a_{max}$ are the inner and upper bounds initialized by 1 and 11, respectively, with a step increase $\delta_{a}$ equal to 1, and *v* shows the spatial resolution of the input image, which leads to 11 thinning and 11 thickening operations for each feature of EAP. Considering the resolution of the image *v* in meters, for an image with a spatial resolution of 1 m per pixel, each profile covers regions in the range of 1000–11,000 m${}^{2}$, which might be a reasonable range of sizes of different urban structures in remote sensing images. The standard deviation is adjusted with respect to the mean of the individual features since the standard deviation shows dispersion from the mean [33]. Therefore, $\lambda_{s}$ is initialized to cover a reasonable amount of deviation, which is mathematically given by:

(5) $$\lambda_{s}\left(PC_{i}\right)=\frac{\mu_{i}}{100}\left\{\sigma_{min},\sigma_{min}+\delta_{s},\sigma_{min}+2\delta_{s},...,\sigma_{max}\right\}$$

where $\mu_{i}$ is the mean of the *i*-th feature and $\sigma_{min}$, $\sigma_{max}$ and $\delta_{s}$ are the inner bound, the upper bound and the step size, respectively, which are set as 2.5%, 27.5% and 2.5% based on experience. The EAP for the standard deviation includes 11 thinning and 11 thickening operations. Figure 2 illustrates the general architecture of EMAP. In this paper, we only use the area attribute and the standard deviation and adopt the above parameter-setting methods.

For the cluster of each pixel in the feature space, existing uncertainty of the number of categories and the massive overlap of feature spaces of different categories, the pixels belonging to different categories cannot be absolutely separable by sharp boundaries. Therefore, a fuzzy clustering technique [9,34] is more appropriate to separate overlapping clusters. In our problem, we assume that a pixel can belong to multiple different categories with certain degrees of membership due to no clear boundary between them. In this study, we choose fuzzy c-means clustering (FCM) to process the above EMAP features. After extracting EMAP features for each pixel, FCM is applied to cluster each pixel in feature space.

### 2.2. Super-Pixel Segmentation and Hierarchical Fuzzy Histogram Construction

There are crucial challenges in the clustering process, i.e., how to predefine a suitable clustering number. We propose a strategy called hierarchical fuzzy clustering in order to avoid the effects of the improper selection of clustering number. For HR remote sensing images, we consider 8 classes as the maximum number of clusters because more clusters did not show an increase in discriminative information and introduced more error classification. Extensive experiments on clustering number selection can be found in Section 3.2.

Given two coregistered multitemporal images $I_{1}$ and $I_{2}$, the FCM clustering is applied to EMAP features in $I_{1}$ and $I_{2}$, where the clustering numbers range from 2 to the maximum number of clusters (i.e., 8). The rationale of this method is to adaptively generate a model of the class spaces of each pixel according to the hierarchical fuzzy clustering strategy. Higher spectral variation and mixed pixels on these HR data lead to the diversity of pixels in the same class and the similarity of pixels across different classes. Considering that, hierarchical fuzzy clustering can be the best solution to this problem. It is more appropriate and realistic to separate overlapping clusters. Each cluster level can distinguish corresponding class space information. Furthermore, EMAPs have been proven to be suitable for extracting spatial information while preserving the geometrical characteristics of the structures and representing the multiscale variability of the structures in the image. The combination of EMAPs and hierarchical fuzzy clustering can preferably identify each pixel for HR images.

Pixel-based strategies lead to generating noises, like isolated changed pixels, holes in the connected changed components or jagged boundaries. Misregistration between multitemporal images is a another critical source of errors. These situations are more obvious for HR images. Characterizing image-objects is less sensitive to the above errors than traditional pixel-based approaches are, which provides great opportunities to better monitor land cover changes than using spectral information alone. Major object-based strategies contain the following two methods: (1) extract object-based features (e.g., geometry, texture and context); (2) derive image-objects by segmentation. The former one is still impossible to independently generate exactly image-object boundaries on account of the nature that pixels are the basic unit of image comparison. The latter one will face difficulty of the selection of segmentation scale, as well. Traditional segmentation algorithms strive to segment out the integrated geographical objects. However, the performance of CD is also strongly influenced by the segmentation algorithms, and the extraction of object boundaries poses a great challenge to segmentation algorithms.

In this paper, a superpixel segmentation algorithm called SLIC proposed by Radhakrishma Achanta [25] is utilized to address the above problems. This method has the following advantages: (1) superpixels adhere well to object boundaries; (2) computational complexity of this method is not high, and the computing speed is fast; (3) the segmentation scale is appropriate. The object size derived from traditional segmentation methods is larger than that from SLIC. Consequently, the distortion of object boundaries derived from conventional methods is greater than that of SLIC, especially for object-based methods, because misclassification of larger objects results in the wrong labeling of more pixels in the final results.

Images acquired from two different dates rarely capture the landscape surface in the same way due to variations of illumination conditions, view angles and meteorological conditions. Thus, objects obtained by separate segmentation on the same site from different images often vary geometrically. Instead, a multitemporal segmentation method is applied in this paper. Firstly, a composite image consisting of all bands of the two images is created by concatenating the pixels along the spectral dimension. Then, PCA transformation is applied, and the first few principle components (the first three ones are selected in our experiments) are extracted, as they contain most of the information (including changed and unchanged ones) in the two images. We call this image the PC image. At last, the PC image is partitioned into compact homogeneous objects with similar spectra. After that, the fuzzy histogram is constructed for each segment by accumulating the degree of membership of the pixel to clusters. The histogram should be normalized by dividing the sum over all of its elements [35]. Then, all of the fuzzy histograms for the clustering numbers ranging from 2 to the maximum number of clusters are catenated to construct a hierarchical fuzzy histogram, which is used to represent this object, i.e., the object feature $F_{l}\left(l\leq L,L\;\text{is}\;\text{the}\;\text{maximum}\;\text{of}\;\text{segments}\right)$ is represented as:

(6) $$F_{l}=\left\{{\tilde{\mu }}_{l1}^{\left(2\right)},{\tilde{\mu }}_{l2}^{\left(2\right)},{\tilde{\mu }}_{l1}^{\left(3\right)},{\tilde{\mu }}_{l2}^{\left(3\right)},{\tilde{\mu }}_{l3}^{\left(3\right)},...,{\tilde{\mu }}_{l1}^{\left(c\right)},{\tilde{\mu }}_{l2}^{\left(c\right)},...,{\tilde{\mu }}_{lc}^{\left(c\right)}\right\}$$

and:

(7) $${\tilde{\mu }}_{lj}^{\left(i\right)}=\frac{\sum_{t=1}^{N}\mu_{tj}^{\left(i\right)}}{\sum_{t=1}^{N}\mu_{t1}^{\left(i\right)}+\sum_{t=1}^{N}\mu_{t2}^{\left(i\right)}+...+\sum_{t=1}^{N}\mu_{tj}^{\left(i\right)}+...+\sum_{t=1}^{N}\mu_{ti}^{\left(i\right)}},\left(1\leq j\leq i\right)$$

where ${\tilde{\mu }}_{lj}^{\left(i\right)}$ means the normalized sum of the degree of belonging of each pixel within the *l*-th object (*N* is the number of pixels in this object) to the *j*-th cluster when the clustering number is *i* (normalized according to (7)). Analogously, $\mu_{tj}^{\left(i\right)}$ means the degree of belonging of the *t*-th pixel to the *j*-th cluster when the clustering number is *i*. This kind of representation allows one to capture and exploit the entire information presented in the considered objects. The traditional bag of words (BOW) approach [36,37,38] constructs the codebook using the k-means algorithm, which is inadequate to capture the abundant spectral information and complex structure in HR images. Similar to BOW, our method establish the codebook by adopting hierarchical fuzzy clustering, which can model preferable spectral and spatial information. The final change features are formed by differencing the corresponding hierarchical fuzzy histograms at two different time instances, $t_{1}$ and $t_{2}$.

### 2.3. Saliency and MBI for Final Change Detection

After obtaining the change features, how to define a decision function that distinguishes changes in unsupervised CD is of great importance. One common approach is applying an empirical threshold value, which is used in most of unsupervised CD algorithms. Another widely-used method is using the Gaussian mixture distribution (GMD) to model the distribution of the features and separate the changed from unchanged class by maximizing a posterior probability. Nonetheless, mis- or over-detection is a common occurrence because of the overlap of distributions of the changed and unchanged class. Considering the complex statistical distributions of the change features, we propose a new semi-supervised classification for CD.

In this paper, saliency detection combined with MBI is utilized to obtain a pseudo training set. This set includes the most reliable samples for the changes or no changes. Saliency computation is an important method to detect the region of interest. It has been widely adopted in many applications like object segmentation and detection. Usually, informative regions that represent the main contents of an image can be selected by saliency computation. In remote sensing images, buildings stand out from their surroundings and draw more attention of people. Meanwhile, the changed regions are also salient across the bi-temporal images. Therefore, saliency detection could be used as a powerful tool for CD. Before extracting the salient regions, we adopt an object-based CVA [16] to generate the difference image instead of traditional pixel-based CVA in order to ensure the consistency within the image-objects, because the difference between pixels in the same object should be comparatively small and the saliency cues between them should be fairly approximate or equal. The object-based change magnitude within the object $R_{l}$ can be represented as $D_{R_{l}}$:

(8) $$D_{R_{l}}=\sqrt{\sum_{i=1}^{b}{\left(\frac{\sum_{x\in R_{l}}I_{1}^{i}\left(x\right)}{N_{1}^{R_{l}}}-\frac{\sum_{y\in R_{l}}I_{2}^{i}\left(y\right)}{N_{2}^{R_{l}}}\right)}^{2}}$$

where *b* is the number of spectral bands, $I_{1}^{i}$ and $I_{2}^{i}$ are respectively the magnitude of image $I_{1}$ and $I_{2}$ at the *i*-th band and $N_{1}^{R_{l}}$ and $N_{2}^{R_{l}}$ denote the corresponding number of pixels in the object $R_{l}$.

Next, we obtain the saliency map from the difference image using the spectral residual approach [26]. This method is efficient, independent of features, categories or other forms of prior knowledge of the objects. Following that, we set a threshold to get two binary images $M_{s}$ and $M_{u}$ marking the most salient regions and the least salient ones, respectively.

Furthermore, this paper mainly focuses on urban building changes. The recently-developed MBI [19,20,35] is able to indicate the presence of buildings in HR images, so it is more helpful for locating changed buildings. The basic idea of MBI is to represent the spectral-structural characteristics of buildings by a set of morphological operators. The simple delineation is as follows,

1. Calculation of brightness: The maximum value of multispectral bands for each pixels is denoted as:
  (9) $$v\left(t\right)=\max_{1\leq i\leq b}\left(band_{i}\left(t\right)\right)$$
  where $band_{i}\left(t\right)$ indicates the intensity of the *t*-th pixel for the *i*-th band.
2. Calculation of ${\text{DMP}}_{\text{TH}}$: Top-hat transformation is able to emphasize the locally bright structures. Additionally, buildings have high local contrast comparing with their spatially adjacent shadows. Therefore, the spectral-structural characteristics of buildings can be represented using the differential morphological profiles (DMPs) [27] of top-hat transformation with multiscale and multidirectional SE, i.e.,
  (10) $$\begin{array}{cc} DMP_{TH} & =|TH_{v}\left(d,s+\Delta s\right)-TH_{v}\left(d,s\right)|\\ TH_{v}\left(d,s\right) & =v-\gamma_{v}^{re}\left(d,s\right) \end{array}$$
  where $TH_{v}\left(d,s\right)$ indicates the top-hat transformation with *d* and $s(s_{min}\leq s\leq s_{max})$ being the direction and scale of a linear SE, respectively, $\gamma_{v}^{re}$ represents the opening by reconstruction of the brightness *v* in (9) and Δs is the interval of the profiles.
3. Calculation of MBI: The MBI is calculated by the following formula
  (11) $$MBI=\frac{\sum_{d}\sum_{s}DMP_{TH}\left(d,s\right)}{D\times S}$$
  where *D* and *S* are the total of directionality and scale. We consider four directions (i.e., 45${}^{\circ }$, 90${}^{\circ }$, 135${}^{\circ }$ and 180${}^{\circ }$) and eleven scales (i.e., $s_{min}=2$, $s_{max}=52$ and Δs=5).

Analogously, we extract MBI image $M_{b}$, which indicates the change information of buildings from the above-mentioned difference image. With the purpose of extracting the most salient building objects, we extract the image-objects that overlap the most with $M_{s}$ and $M_{b}$ as the changed class. Considering that the unchanged class is not generally salient in difference images and includes buildings and non-buildings, we extract the ones overlapping the least only with $M_{u}$ as the unchanged class. The changed and the unchanged objects constitute the pseudo training set. Finally, RF is used to classify the object-specific change features with the aforementioned training samples, and the final CD results are obtained.

## 3. Results and Discussion

### 3.1. Datasets

In order to assess the effectiveness of the proposed method, to begin with, we conduct the experiments on a pair of images used in [16] as shown in Figure 7a,b. These images are taken over Beijing, acquired by QuickBird in September 2002 and November 2003, and cut into 472×472 pixels. The second dataset used in our experiments is cropped from Google Earth. It contains five pairs of bi-temporal images over the Beijing urban area. See those images in Figure 8a,b. These images have only three bands in the visible spectrum with a fixed size of 500×500. The typical image registration is executed as the basic pre-processing step. Nevertheless, the corresponding images from different times have a diversity of spectral colors. With the rapid infrastructure construction and updating, the datasets show complicated land cover changes. The first, third and fourth pairs are taken on 30 September 2012 and 4 March 2013. The second pairs are on 28 June 2009 and 19 September 2015. The spatial resolution of the first four pairs is 1 m. The last pairs are acquired on 4 March 2013 and 12 November 2014, with a spatial resolution of 4 m.

### 3.2. Experiments

Seven widely-used methods are chosen to be compared, including the EM-based method [5], the MRF-based method [5], the PCA-based method [7], the parcel-based method [15], the MBI-based method [19], the sparse hierarchical clustering (SHC)-based method [21] and the fast object-level-based method [16].

1. Evaluation indexes:
  Five indexes are used to evaluate the accuracy of above-mentioned methods.
  - False alarms (FAs): the number of unchanged pixels that are incorrectly detected as changed ones, i.e., $N_{FA}$. The false alarm rate (FAR) is calculated as $R_{FAR}=\frac{N_{FA}}{N_{0}}\times 100\%$, where $N_{0}$ is the total number of unchanged pixels;
  - Missed alarms (MAs): the number of changed pixels that are incorrectly detected as unchanged ones, i.e., $N_{MA}$. The missed alarm rate (MAR) is calculated as $R_{MAR}=\frac{N_{MA}}{N_{1}}\times 100\%$, where $N_{1}$ is the total number of changed pixels;
  - Overall alarms (OAs): the total number caused by FAs and MAs; the overall alarm rate (OAR) is calculated as $R_{OAR}=\frac{N_{FA}+N_{MA}}{N_{0}+N_{1}}\times 100\%$;
  - Kappa coefficient (kappa): the consistency between experimental results and the ground truth; it is expressed as $kappa=\frac{P_{o}-P_{c}}{1-P_{c}}$, where $P_{o}$ indicates the real consistency and $P_{c}$ indicates the theoretical consistency.
2. Parameter setting:
  The approaches used for comparison are implemented using the same set of parameters presented in their related papers. The EM-based method is free of parameters. The MRF-based method depends on the parameter *β*, which tunes the influence of the spatial contextual information, and we selected β=4. The PCA-based method has two parameters, i.e., non-overlapping blocks *h* (h=4 in our experiments) and the dimensions *S* (S=3 in our experiments) of the eigenvector space. In the parcel-based method, the parameters in hierarchical segmentation are tuned to achieve the best performances as [15]. The MBI-based method is implemented as [19] where the thresholds of the spectral condition, the MBI condition, the area and the geometrical index are respectively 0.3, 0.2, 30 and 2.0. In the SHC-based method, we adopt the parameter setting the same as [21]. For the fast object-level based method, the parameter setting we used is also the same as [16].

**Figure 3:**
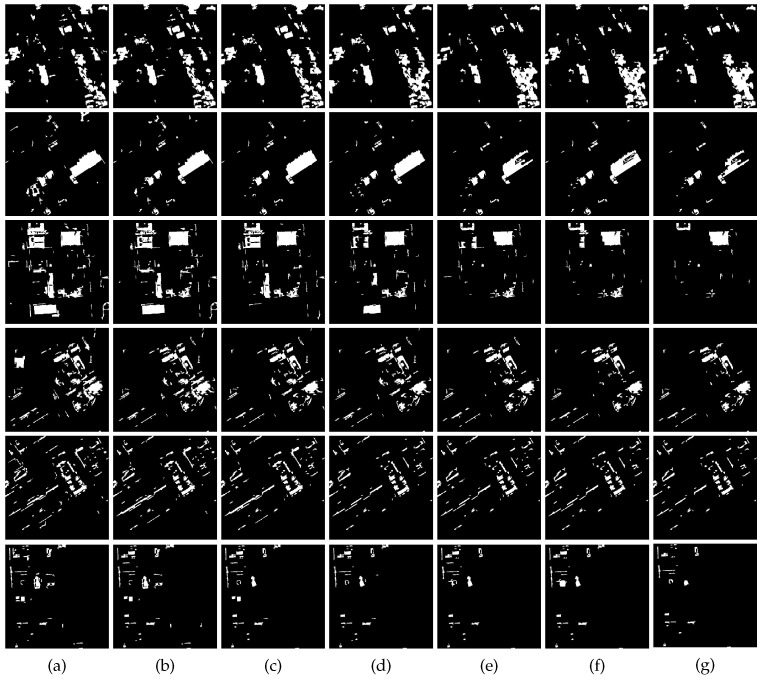
The influence of different clustering numbers. From top to bottom: corresponding results of the aforementioned two sets of image pairs; from left to right: different clustering numbers. (**a**) 2–4; (**b**) 2–6; (**c**) 2–8 (used in our experiments); (**d**) 2–10; (**e**) 2–12; (**f**) 2–14; (**g**) 2–16.

**Figure 4:**
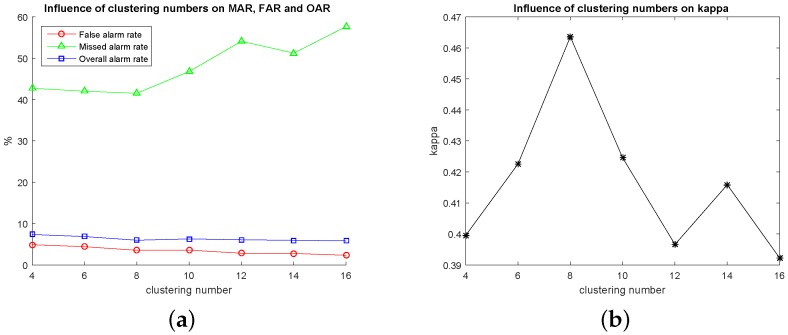
The influence of different clustering numbers. The clustering numbers range from 2–4 to 2–16. (**a**) Influence of clustering numbers on false alarm rate (FAR), missed alarm rate (MAR) and overall alarm rate (OAR); (**b**) influence of clustering numbers on kappa.

**Figure 5:**
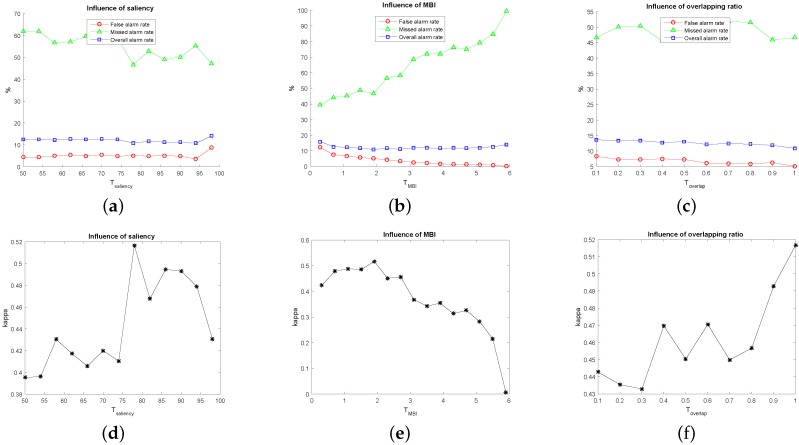
Sensitivity delineation of the parameters for the first image pair. (**a**) Influence of saliency on FAR, MAR and OAR; (**b**) influence of MBI on FAR, MAR and OAR; (**c**) influence of overlapping ratio on FAR, MAR and OAR; (**d**) influence of saliency on kappa; (**e**) influence of MBI on kappa; (**f**) influence of overlapping ratio on kappa.

**Figure 6:**
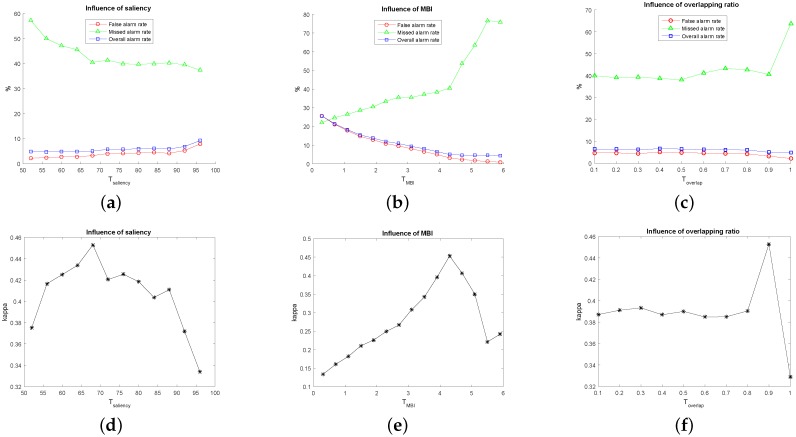
Sensitivity delineation of the parameters for the second image pairs. (**a**) Influence of saliency on FAR, MAR and OAR; (**b**) influence of MBI on FAR, MAR and OAR; (**c**) influence of overlapping ratio on FAR, MAR and OAR; (**d**) influence of saliency on kappa; (**e**) influence of MBI on kappa; (**f**) influence of overlapping ratio on kappa.

For our method, EMAPs are constructed using the area attribute and the standard deviation as given in Section 2.1. In our experiments, for each image, 69 dimension EAPs on the area attribute and 66 dimension EAPs on the standard deviation were generated, i.e., 135 dimension EMAPs. The clustering number of hierarchical fuzzy clustering is eight as previously mentioned. Considering the complexity of our used datasets, we adopt 16 to serve as the maximum clustering number for comparisons. Figure 3 shows the qualitative results, which present the change maps with different image pairs and clustering numbers, and Figure 4 shows the quantitative results, which present the influence of different clustering numbers on MAR, FAR, OAR and kappa. From Figure 3, it can be seen that there will be more false detection areas when the clustering number decreases, and there will be more missed detections when it increases. From Figure 4, we can see that MAR reaches the minimum value, and kappa reaches the optimum value when the clustering number is eight. It can be seen that the best results are obtained when the clustering number is eight. As for the segmentation method SLIC, two parameters need to be selected: the nominal size of the regions and the strength of the spatial regularization. The former one is used to control the size of the image grid for division. The latter one sets the trade-off between clustering appearance and spatial regularization. In this paper, 30 and one are selected for them based on experience. Other parameters, including threshold $T_{saliency}$ for saliency map, $T_{MBI}$ for MBI image and overlapping ratio $T_{overlap}$ between building regions and salient regions, are determined to obtain the best results. The influence of $T_{saliency}$, $T_{MBI}$ and $T_{overlap}$ on FAR, MAR, OAR and kappa is shown in Figure 5 and Figure 6. When we analyze the sensitivity of each parameter, the other parameters are set to be the constant optimal values.

From Figure 5, it can be seen that FAR and OAR tend to be stable, and MAR has slight fluctuations as the saliency increases. When $T_{saliency}=78$, kappa is improved significantly. It is demonstrated that saliency plays a vital role. With $T_{saliency}$ increasing, it also restricts the performance due to the insufficiency of the training samples. In addition, MAR also reduces to the lowest value. Increasing of $T_{MBI}$ results in the gradual increase on MAR and the decrease on FAR and OAR. Additionally, when $T_{MBI}=1.9$, we can get a tradeoff between MAR, FAR and OAR, also the best results on kappa, as shown in Figure 5e. The performance decreases when $T_{MBI}$ gets smaller or larger than 1.9. The influence of the overlapping ratio on MAR, FAR and OAR is not obvious. However, there appears some fluctuations when $T_{overlap}$ are between 0.3 and 0.7. After that, kappa rises steadily and reaches the optimal value when $T_{overlap}=1$. Higher values in $T_{overlap}$ filter out incorrect samples and improve the classification results.

As for Figure 6, with the increasing value of $T_{saliency}$, FAR and OAR are relatively stable, but MAR decreases significantly and reaches a relatively low value when $T_{saliency}=68$. kappa increases gradually and reaches the peak point around $T_{saliency}=68$. Over this point, it decreases continually and has a slight fluctuation when $T_{saliency}$ is between 70 and 90 because the guidance of saliency is unstable in this range. Finally, it descends rapidly due to the reduction of the correct training samples caused by the excessively strict saliency limitation. This models can achieve the best performance on this point. It can be seen that both too high and too low values lead to the introduction of the wrong samples, which reduces the detection accuracies. As for MBI, in the beginning, the increase of MBI will result in the increased kappa and the decreased OAR and FAR. The results achieve the optimum when MBI reaches the appropriate threshold. After that, kappa decreases dramatically, and MAR rises sharply. It is observed that MBI is also of key importance, the same as saliency. By analyzing the results with different percentages for overlapping, it can be seen that the increase of overlapped ratio is insensitive to the detection performance. It mainly lies in that the object-based CVA reduces the heterogeneity within each block, which results in the characteristics of each pixel within the same block being essentially consistent. FAR, OAR and kappa reach the optimum when $T_{overlap}=0.9$, but the performance is not good when $T_{overlap}=1$. It demonstrates that too strict limits seem adverse to the performance.

On the whole, our approach has relatively high MAR. The first image pair has low resolution. The second image pairs not only have higher spatial resolution, but also have more complicated geographic structures and spectral intensity. Considering the difference of the radiance, spatial resolution and structure of geographic objects, the curve shapes of the two sets are different. Therefore, based on the above discussions, for the first set of image pairs, $T_{saliency}=78$, $T_{MBI}=1.9$ and $T_{overlap}=1$ are used, and for the second ones, $T_{saliency}=68$, $T_{MBI}=4.3$ and $T_{overlap}=0.9$ are adopted.

### 3.3. Results and Analyses

**Figure 7:**
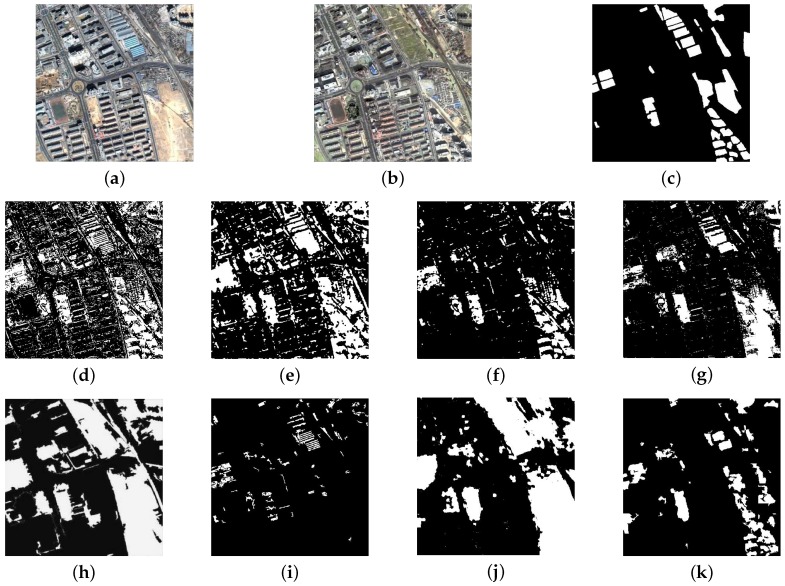
The first image pairs and the results of different methods. (**a**) Image in 2002; (**b**) image in 2003; (**c**) ground truth; (**d**) EM-based; (**e**) Markov random field (MRF)-based; (**f**) PCA-based; (**g**) parcel-based; (**h**) fast object-level; (**i**) MBI-based; (**j**) sparse hierarchical clustering (SHC)-based; (**k**) proposed.

We designed the experiments on the above two sets of image pairs to validate the effectiveness of our approach. On the first pair images, the performance of the proposed CD algorithm is compared with seven approaches. Figure 7 shows comparison results of the input images $I_{1}$ and $I_{2}$ (see Figure 7a,b). The corresponding reference change map is shown in Figure 7c. Figure 7d–k shows the CD results of the EM-based method, the MRF-based method, the PCA-based method, the parcel-based method, the fast object-level method, the MBI-based method, the SHC-based method and the proposed method. The quantitative performances are listed in Table 1.

The EM-based method and the MRF-based method generate more scattered changed areas. This poor performance mainly lies in the limitation of traditional pixel-based image analysis and the multimodal distribution of the change feature. The number of overall alarms is reduced, attributed to the use of MRF-based contextual information. The PCA-based method uses block-based data analysis to import the local neighborhoods, which reflect the contextual information. Therefore, it decreases incorrect detection and increases missed detection. The parcel-based method exploits the multitemporal and spatial contexts at different scales based on hierarchical segmentation. Its overall alarms are slightly higher than the PCA-based method, but it has a better kappa. For the fast object-level method and the SHC-based method, all of them can generate massive changed areas avoiding making fragmentary areas. However, they bring in too much changed areas, including bare soil and vegetation, etc. Although the MBI-based method is able to indicate the presence of buildings using MBI, it is sensitive to scales and spectral changes. As a consequence, it may loss abundant building areas and only detects a fraction of changed ones and yields high MAR. The result of the proposed technique indicates the best qualitative and quantitative results compared with other approaches. Our method detects most of the changed building areas. It has the same lower overall alarms as the PCA-based method does. However, certain areas may be missed, which is a drawback of this method. From the table, we can conclude that our approach is superior to other methods in terms of OAR and kappa, except for relatively high MAR.

**Figure 8:**
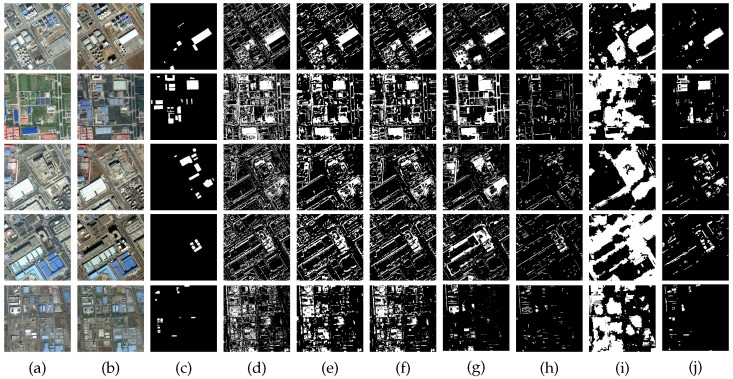
The second image pairs and comparisons of different methods. From top to bottom: five image pairs (Image 1–Image 5). (**a**,**b**) Images from two different times; (**c**) ground truth; (**d**) EM-based; (**e**) MRF-based; (**f**) PCA-based; (**g**) parcel-based; (**h**) MBI-based; (**i**) SHC-based; (**j**) proposed.

We further conduct extensive experiments on more challenging images collected from Google Earth, which have more complex spectral information, as shown in Figure 8a,b. The manually-delineated ground truth maps are presented in Figure 8c. The proposed approach is compared against the six above-mentioned approaches, that is EM-based, MRF-based, PCA-based, parcel-based, MBI-based and SHC-based. The former three methods produce more false changed areas. They are excessively sensitive to the changed shadows and spectral differences caused by illumination and sensors and susceptible to displacement caused by registration errors, of which the EM-based method is the most serious. It is obvious that traditional pixel-based strategies, such as the EM-based method, are less effective for HR images. The introduction of spatial-contextual information improves the results to some extent. The MRF-based method exploits inter-pixel class-dependent contexts. The PCA-based method considers the spatial context by extracting eigenvectors on the non-overlapping blocks of the difference image applying PCA. However, its square window of a fixed size limits its performance, and the blocks localized on the boundaries between changed and unchanged regions bring about some error detection. The parcel-based method is relatively robust and has fewer false changed areas. It has good performance by analyzing multilevel and multitemporal parcel-based context information, but it still cannot avoid the defect of pixel-based methods for HR images. The MBI-based method is good at detecting small-scale building changes, as shown in the fourth row of Figure 8h. However, it ignores large-scale building changes. For the SHC-based method, it can find almost all of the possible changed areas, consequentially leading to more false changed areas.

The superiority of our approach could be seen from Figure 8j. The EMAPs and the hierarchical fuzzy histogram improve the discriminative ability and the robustness of the features. Our approach mainly yields partial missed changed areas (see Images 2 and 3 of Figure 8j) and only fewer false ones (see Images 4 and 5 of Figure 8j). In Image 2 of Figure 8j, the several buildings on the left are not detected due to the insensitivity of these kinds of spectral differences, and the missed detections of Image 3 are on account of the spectral similarity before and after the changes. In Image 4 of Figure 8j, the false detections are mainly derived from the effects of misregistration and shadow. The buildings of Image 5 located in the upper left corner are spurious changes generated by the spectral difference caused by illumination. The mistakes of MBI detection and the high spectral reflection give rise to the false water detection of Image 5.

The quantitative accuracies of the different methods are in Table 2. From the table, it is apparent that our approach, with fewer OAR and kappa, produces better CD results than other methods. The only disadvantage is relatively high MAR. Compared with other methods, our method on MAR ranks in the bottom half, which performs better than the parcel-based method and the MBI-based method generally and better than the PCA-based method in a few cases. The possible reasons are mainly the inaccuracy of saliency and MBI. The EM-based method performs the poorest on almost all of the images because of the excessively simple difference operator and threshold selection. The MRF-based method reduces MAR and FAR compared with the EM-based method due to the spatial contextual information. It even outperforms the PCA-based method and the parcel-based method in some images. The PCA-based method and the parcel-based method have relatively low OAR and high kappa attributed to the spatial information introduced by different partitioning strategies. The MBI-based method performs well on the images of a relatively small scale, such as Images 4 and 5. The SHC-based method is prone to have high FAR and results in better performance in the images that have small color differences, such as Images 1 and 3.

**Figure 9:**
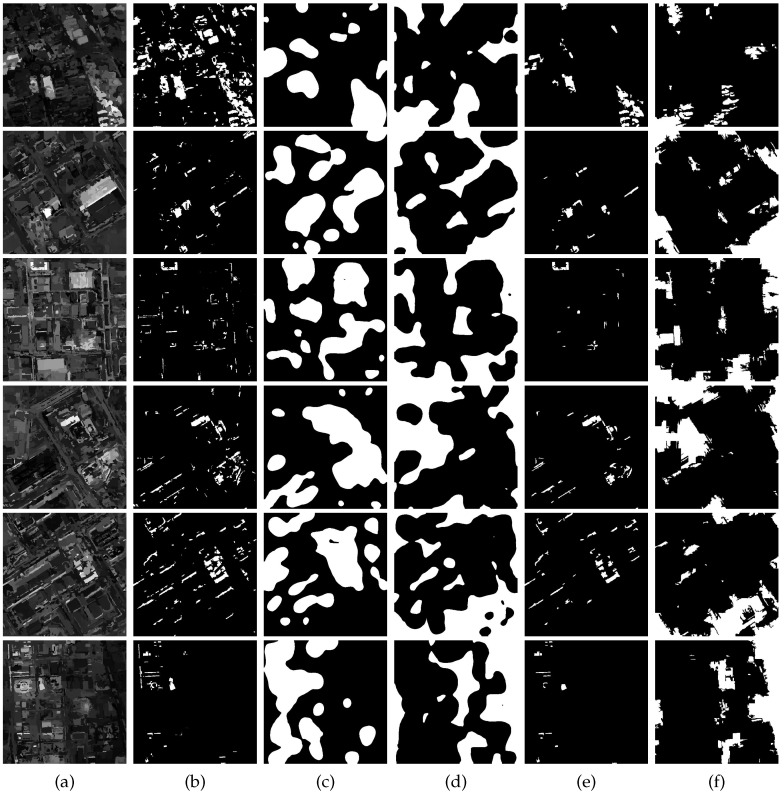
Intermediate results of MBI and saliency. From top to bottom: corresponding results of the two aforementioned datasets. (**a**) The difference image by object-based CVA; (**b**) building regions by thresholding MBI. (**c**) salient regions; (**d**) non-salient regions; (**e**) changed objects of training sets; (**f**) unchanged objects of training sets.

Our approach not only adopts the feature extraction methods of EMAPs and the hierarchical fuzzy histogram, but also utilizes a semi-supervised strategy that we select some potential training samples by means of the combination of saliency and MBI for the final refined classification. To clearly describe this strategy, the intermediate results for the six above-mentioned datasets are reported in Figure 9. Figure 9a shows the difference image by object-based CVA. It can be seen that the changed areas have higher brightness values and that of the unchanged ones are decreased, and this operation preserves the contours and edges of the buildings and generates higher homogeneity within each object, which is beneficial to the calculation of MBI and saliency. Figure 9b represents the possible changed building areas extracted by MBI. From the figure, we can see that there are many missed areas and false ones, but the primary building areas still could be detected. Saliency detection on difference images can locate the apparent changed areas and unchanged ones in spite of the production of error detection, as shown in Figure 9c,d, which plays an important role as a guidance for generating the pseudo training sets. The combination of saliency and MBI filters out plenty of false detected areas and reserves the most crucial changed ones for the subsequent classification, as shown in Figure 9e. Figure 9f demonstrates the unchanged areas derived from the non-salient detection of Figure 9d. We take advantage of potential changed areas (Figure 9e) and unchanged ones (Figure 9f) as the training sets for RF and transform the original unsupervised method to the supervised one, so as to effectively improve the performance for CD, as shown in Figure 8j. From Figure 9e,f, it can be seen that our constructed features are extremely discriminative, because we only use a few training sets and achieve good performances. In all cases, the proposed method outperforms all of the other methods over both qualitative and quantitative measures.

## 4. Conclusions

In this paper, a novel CD approach for HR remote sensing images is presented. The proposed method combines pixel-based post-classification with object-based semi-supervised classification and achieves a promising performance on challenging datasets.

In the stage of pixel-based post-classification, we adopt recently-developed EMAPs for the feature extraction of each pixel, which is frequently used for multispectral and hyperspectral image classification. Then, we propose a hierarchical fuzzy histogram construction for the feature extraction of each object, which is obtained by super-pixel segmentation SLIC. This enables the sufficient integration of the pixel-based and object-based advantages. Our approach not only preserves the spectral characteristic of each pixel avoiding the loss of information, but also solves the limitation of the CD methods only depending on the analysis of the single pixel for HR images by introducing the object level strategy.

As for the stage of object-based semi-supervised classification, we propose a novel strategy, which is to acquire the most possible changed building areas and unchanged areas as the input of RF by utilizing saliency detection and MBI. The advantages of supervised CD methods are making the best use of these, and the disadvantages of unsupervised CD methods are adequately avoided.

Compared with a variety of CD methods, our proposed approach is promising in robustness and effectiveness. Moreover, the qualitative and quantitative results show that the proposed approach produces fewer OAR and higher kappa for the HR images in our experiments. Despite the comparable results achieved by the proposed method, there are still many improvements to consider in the future work. Firstly, we should deliberate on the reduction of the MAR of our approach. Excessively high MAR is a drawback of our method. It may be helpful for solving this problem to add some a priori information to obtain more reliable and representative samples. For example, shadow information can be used as a constraint to identify buildings and used to eliminate the spurious changes caused by shadow, and the vegetation index can be used to extract tree and grass backgrounds to reduce the mistakes further. Then, from the experiments, it is seen that our approach cannot avoid a few false detections caused by the spectral difference of the same class. In subsequent work, we consider adding some preprocessing steps to solve this, such as the transformation and normalization of the color space. In addition, how to extract more discriminative features and adopt more robust difference measures should be considered for the object-based change feature representation. Furthermore, automatic selection of the parameters should be the focus of the latter research.

## Figures and Tables

**Table 1:** Performance comparisons against different approaches on the first set of image pairs.

Accuracy		EM-Based	MRF-Based	PCA-Based	Parcel-Based	Fast Object-Level	MBI-Based	SHC-Based	Proposed
Total Pixels	Changed	31,198	31,198	31,198	31,198	31,198	31,198	31,198	31,198
	Unchanged	191,586	191,586	191,586	191,586	191,586	191,586	191,586	191,586
False Alarms		43,113	40,298	12,505	19,347	51,372	**3,672**	47,912	9,711
		(0.2250)	(0.2103)	(0.0653)	(0.1010)	(0.2681)	**(0.0192)**	(0.2501)	(0.0507)
Missed Alarms		11,840	7,497	16,647	13,379	**2,197**	26,132	4,337	14,543
		(0.3795)	(0.2403)	(0.5336)	(0.4288)	**(0.0704)**	(0.8376)	(0.1390)	(0.4662)
Overall Alarms		54,953	47,795	29,152	32,726	53,569	29,804	52,249	**24,254**
		(0.2467)	(0.2145)	(0.1309)	(0.1469)	(0.2405)	(0.1338)	(0.2345)	**(0.1089)**
kappa		0.2786	0.3815	0.4247	0.4353	0.3985	0.2050	0.3855	**0.5167**

**Table 2:** Performance comparisons against different approaches on the second set of image pairs. (Image 1–Image 5 respectively correspond to five image pairs from top to bottom in Figure 8).

Dataset	Accuracy		EM-Based	MRF-Based	PCA-Based	Parcel-Based	MBI-Based	SHC-Based	Proposed
Image 1	Total Pixels	Changed	11,613	11,613	11,613	11,613	11,613	11,613	11,613
		Unchanged	238,387	238,387	238,387	238,387	238,387	238,387	238,387
	False Alarms		44,974	32,915	33,189	31,828	11,855	52,964	**4,379**
			(0.1887)	(0.1381)	(0.1392)	(0.1335)	(0.0497)	(0.2222)	**(0.0184)**
	Missed Alarms		1,507	855	1,464	1,913	8,394	**312**	1,836
			(0.1298)	(0.0736)	(0.1261)	(0.1647)	(0.7228)	**(0.0269)**	(0.1581)
	Overall Alarms		46,481	33,770	34,653	33,741	20,249	53,276	**6,215**
			(0.1859)	(0.1351)	(0.1386)	(0.1350)	(0.0810)	(0.2131)	**(0.0249)**
	kappa		0.2451	0.3408	0.3195	0.3154	0.1992	0.2379	**0.7458**
Image 2	Total Pixels	Changed	22,402	22,402	22,402	22,402	22,402	22,402	22,402
		Unchanged	227,598	227,598	227,598	227,598	227,598	227,598	227,598
	False Alarms		84,846	55,480	61,075	63,925	15,245	98,316	**9,343**
			(0.3728)	(0.2438)	(0.2683)	(0.2809)	(0.0670)	(0.4320)	**(0.0411)**
	Missed Alarms		9,505	7,240	11,449	11,489	18,878	**3,632**	10,788
			(0.4243)	(0.3232)	(0.5111)	(0.5129)	(0.8427)	**(0.1621)**	(0.4816)
	Overall Alarms		94,351	62,720	72,524	75,414	34,123	101,948	**20,131**
			(0.3774)	(0.2509)	(0.2901)	(0.3017)	(0.1365)	(0.4078)	**(0.0805)**
	kappa		0.0806	0.2197	0.1104	0.1004	0.0974	0.1397	**0.4917**
Image 3	Total Pixels	Changed	14,347	14,347	14,347	14,347	14,347	14,347	14,347
		Unchanged	235,653	235,653	235,653	235,653	235,653	235,653	235,653
	False Alarms		49,440	39,160	39,461	40,730	10,775	66,260	**9,334**
			(0.2098)	(0.1662)	(0.1675)	(0.1728)	(0.0457)	(0.2812)	**(0.0396)**
	Missed Alarms		7,082	6,851	6,398	5,184	11,971	**879**	8,250
			(0.4936)	(0.4775)	(0.4459)	(0.3613)	(0.8344)	**(0.0613)**	(0.5750)
	Overall Alarms		56,522	46,011	45,859	45,914	22,746	67,139	**17,584**
			(0.2261)	(0.1840)	(0.1834)	(0.1837)	(0.0910)	(0.2686)	**(0.0703)**
	kappa		0.1243	0.1732	0.1857	0.2153	0.1248	0.2094	**0.3722**
Image 4	Total Pixels	Changed	3,384	3,384	3,384	3,384	3,384	3,384	3,384
		Unchanged	246,616	246,616	246,616	246,616	246,616	246,616	246,616
	False Alarms		49,500	51,376	38,816	43,902	13,646	133,047	**11,489**
			(0.2007)	(0.2083)	(0.1574)	(0.1780)	(0.0553)	(0.5395)	**(0.0466)**
	Missed Alarms		1,012	738	766	1,093	1,227	**44**	1,094
			(0.2991)	(0.2181)	(0.2264)	(0.3230)	(0.3626)	**(0.0130)**	(0.3233)
	Overall Alarms		50,512	52,114	39,582	44,995	14,873	133,091	**12,583**
			(0.2020)	(0.2085)	(0.1583)	(0.1800)	(0.0595)	(0.5324)	**(0.0503)**
	kappa		0.0620	0.0685	0.0942	0.0689	0.2072	0.0220	**0.2506**
Image 5	Total Pixels	Changed	4,103	4,103	4,103	4,103	4,103	4,103	4,103
		Unchanged	245,897	245,897	245,897	245,897	245,897	245,897	245,897
	False Alarms		57,854	49,947	50,753	14,108	6,840	98,441	**3,895**
			(0.2353)	(0.2031)	(0.2064)	(0.0574)	(0.0278)	(0.4003)	**(0.0158)**
	Missed Alarms		1,153	**808**	1,244	3,516	3,290	812	2,001
			(0.2810)	**(0.1969)**	(0.3032)	(0.8569)	(0.8019)	(0.1979)	(0.4877)
	Overall Alarms		59,007	50,755	51,997	17,624	10,130	99,253	**5,896**
			(0.2360)	(0.2030)	(0.2080)	(0.0705)	(0.0405)	(0.3970)	**(0.0236)**
	kappa		0.0621	0.0871	0.0707	0.0378	0.1195	0.0316	**0.4046**
